# Development and Preliminary Validation of the Couples’ Stigma Scale to Assess Self-Stigma among the Partners of Persons with Autism Spectrum Disorder in Japan

**DOI:** 10.3390/ijerph17103533

**Published:** 2020-05-18

**Authors:** Deguchi Naoko, Asakura Takashi, Omiya Tomoko

**Affiliations:** 1 Public Health Nursing, Faculty of Medicine, University of Tsukuba, Tsukuba 305-8575, Japan; toomiya-tky@umin.ac.jp; 2 Laboratory of Health and Social Behavior, Faculty of Education, Tokyo Gakugei University, Tokyo 184-0015, Japan; asakurat@u-gakugei.ac.jp

**Keywords:** autism spectrum disorder, couples stigma scale, self-stigma, construct validity, scale development

## Abstract

Spouses of individuals with autism spectrum disorder (ASD) may struggle with self-stigma and may require attention and care; however, no scale exists to measure the stigma of spouses of persons with ASD. This study created and investigated the construct validity of the Couples Stigma Scale. This scale consists of 14 items and it was designed based on prior literature, interviews, and the self-stigma theory to assess the self-stigma experienced by spouses of people with ASD. A survey was conducted with spouses of persons with ASD who participated in a self-help group. Responses were obtained from 259 people, of which 253 women were included in the analysis. Exploratory factor analysis was performed separately with two independent groups, indicating a four-factor structure, to determine structural validity. The factor loadings of the items constituting the four factors were 0.39 or greater. Regarding external validity, the correlation coefficient between the Couples Stigma Scale and the World Health Organization Quality of Life (WHOQOL) score was −0.341 (*p* < 0.001), and the domain correlation coefficient was significant for all relevant WHOQOL domains. Our results suggest that the Japanese version of the Couples Stigma Scale is a valid instrument for assessing self-stigma in the spouses of persons with ASD.

## 1. Theoretical Background

Stigma relates to the relationship between a person who suffers a disadvantage and one who disadvantages an individual or group [1]. Recently, research has focused on the self-stigma of families of persons with autism spectrum disorder (ASD), and this issue of self-stigma for those whose spouses have ASD is an urgent topic for study in Japan. A recent study using population-based survey data reported the estimated prevalence of ASD in Japan as being 1.68% [2]. Although prevalence estimates vary somewhat between studies, the rate in Japan is reportedly higher than in some other countries that have been studied [3]. Previously, individuals with ASD were considered awkward in interpersonal relationships, and it was assumed that adults with ASD rarely married [4,5]. Therefore, spouses’ self-stigma has mostly gone unrecognized, and the number of spouses of persons with ASD in Japan has not been clarified. In fact, many high-functioning individuals with ASD have interest and experience in romantic relationships [6] and sexuality [7,8], and the proportion of individuals with ASD who marry has been reported to be 10% [9] to 16% [10]. However, spouses of individuals with ASD are said to struggle with self-stigma, requiring attention and additional care [11]. Spouses of persons with ASD also report discomfort and struggles with loneliness after marriage. Moreover, they describe misunderstandings with others that lead to social exclusion, feelings of being misunderstood, and experiences, such as “being blamed for not living up to the spouse’s role”. Such experiences of the loss of spousal identity and connection with others [12] may be interpreted as stigmatization, yet no measures currently exist for assessing the self-stigma of persons with a spouse with ASD. Therefore, we aimed to develop a spouse self-stigma survey and examine its construct validity in order to assess self-stigma among spouses of individuals with ASD.

### 1.1. Stigma

Link et al. [13] cited four components of stigma: labeling, stereotyping, separating, and emotional reaction. Stigma is defined as negative stereotyping and separation from groups who are labeled as different, which limits access to material, social, and cultural resources for members of stigmatized groups. Corrigan and Watson [14] classified stigma into “public stigma” and “self-stigma”. Public stigma refers to the general public’s stigmatizing of people with mental illness, and self-stigma applies to people with a mental illness who internalize the negative views of society toward themselves. The process of internalizing self-stigma involves negative beliefs regarding the self and agreement with these beliefs, negative emotional reactions, and behavioral responses to prejudice [14]. Boyd and Otilingam [15] developed the Brief Version of the Internalized Stigma of Mental Illness (ISMI) Scale for persons with mental illness. They showed that the internalized stigma of mental illness impedes recovery and it is associated with increased depression, reduced self-esteem, reduced recovery orientation, and reduced empowerment [15].

### 1.2. Self-Stigma of Families

Stigma affects not only individuals with labels, but also those who are closely related to them [16,17]. “Courtesy stigma” is the negative impact of being associated with a person with stigma [16], and “associative stigma” is passed on from stigmatized people to members of their social networks [17]. “Affiliate stigma” refers to the extent of self-stigmatization among associates of the targeted minorities, and its dimensions include affective, cognitive, and behavioral aspects [18].

Some scales exist on the self-stigma of families. The Affiliate Stigma Scale [18] measures self-stigma of the caregivers of people with mental illness and intellectual disability. The Family Stigma Instrument, which measures the self-stigma of family members of people with intellectual disabilities and comorbidities, has also recently been developed [19]. Research is ongoing on families of those with mental illness and intellectual disability.

Previous studies on self-stigma have demonstrated significant negative correlations with psychosocial variables, including quality of life (QOL) [20,21]. The World Health Organization (WHO) defines QOL as an “individual’s perception of their position in life in the context of the culture and value systems in which they live and in relation to their goals, expectations, standards, and concerns” [22]. The scale World Health Organization Quality of Life-BREF(WHOQOL-BREF) contains domains that measure physical, psychological, and social relationships, overall QOL, general health, and the environment [23]. The self-stigma theory suggests that, as a result of internalized stigma, persons experience reduced self-esteem and self-efficacy, which might lead them to avoid pursuing life goals [24]. The avoidance of the pursuit of life goals leads to lower QOL. Given this, self-stigma might be closely related to the concept of QOL.

Stigma may vary depending on the relevant disability [25], and people living with partners with autism in Japan may experience various types of discrimination. For example, caregivers of individuals with ASD experience greater stigma than do caregivers of individuals with intellectual or physical disabilities [25]. Previous studies on self-stigma of families of individuals with ASD show that parents exhibit difficulties regarding self-stigma [26,27], repeated experiences of guilt and remorse [28], lack of knowledge of their surroundings [29], and social exclusion [30]. They may also hide the behavioral characteristics of their children [31] and how they deal with them [32]. Spouses of persons with ASD report misunderstandings with others that lead to social exclusion [12]. However, the processes and theoretical constructs of self-stigma of spouses—rather than parents—of high-functioning individuals with ASD have not been previously studied.

Stigma may also vary, depending on one’s family role [33,34]. Spouses of persons with mental illness are blamed for not ensuring that relatives with mental illness adhere to treatment plans [33]. A study of families (i.e., parents, spouses, children, and siblings) of persons with schizophrenia, drug dependence, or emphysema, demonstrated that their parents and spouses were viewed as being more responsible than their children or siblings for the onset of the person’s schizophrenia, drug dependence, or emphysema [34]. Although it is speculated that parents of children with ASD with intellectual disability and the spouses of individuals with ASD without intellectual disability are fundamentally different, there is no scale currently available to measure spouses’ self-stigma.

Therefore, the goal of our study was to examine the construct validity of a self-stigma scale for ASD spouses. We created this scale based on interviews with spouses of persons with ASD and a review of previous studies.

## 2. Methods

### 2.1. Conceptualization and Development of an Initial Item Pool

We referred to a published method for examining the construct validity of newly created instruments for taking objective measurements [35]. First, we cleared the conceptualization of target constructs through a literature search, qualitative interviews exploring sub-dimensions, and the application of a theoretical model. We extracted the question items, conducted pilot testing, and made corrections based on the advice of two representatives of the self-help group and a scale-creation expert after applying a logical approach for the clear conceptualization of the target constructs. Thus, the Couples Stigma Scale (CSS) item pool was generated through a multi-step process that is described in the following sections.

#### 2.1.1. Literature Search

Clark and Watson [35] highlighted the importance of conducting a thorough literature review at this stage to make sure the proposed scale is distinct from previous efforts, and possible sub-dimensions should also be planned and anticipated. As a study of the concept of self-stigma, we conducted a systematic scoping review aimed at understanding the dimensions of self-stigma that are unique to the families of persons with ASD, and the conditions in which self-stigma in families of persons with ASD arises. First, four electronic databases were searched—Psych INFO, Web of Science, PubMed, and Ichushi—for studies, including dimensions of self-stigma in families of persons with ASD. The shortened final search strategy combined all ASD or its related words, stigma or its related words, and family or its related words. Seventeen studies met our inclusion criteria and provided qualitative information regarding the dimensions of self-stigma. Additional searches were also made in relevant gray literature and by manually studying reference lists of identified articles. However, no study on spouses of people with ASD was found, and only studies on parents of people with ASD existed. We referred to the meta-synthesis method to identify the self-stigma of families with ASD [36,37]. The identified dimensions included social misunderstanding, negative prejudice, social rejection, isolation, emotional reactions, and stigma management. The dimension of social misunderstanding was unique to families of persons with ASD.

#### 2.1.2. Qualitative Interviews to Explore Sub-Dimensions 

We conducted semi-structured interviews (*n* = 13) with the couples of persons with ASD, whose experiences in married life varied, such as the number of years they had been married, the number of children they had, and occupations of the participants and their spouses in order to explore the appropriateness of using the sub-dimensions in the CSS. All of the interviewees were women. The first author conducted the interviews, which lasted 40 min. to 2 h per person. The interviews were conducted face-to-face in a quiet, private room. All of the interviews were recorded on an IC-recorder and then transcribed. The interviews were deductively analyzed, applying a modified version of the grounded theory approach. Four structural concepts were extracted related to the self-stigma process that was revealed through interviews with spouses/partners of persons with ASD. These dimensions were social misunderstanding, awareness of negative gazes, emotions arising from negative gazes, and self-regulatory behavior in order to avoid negative gazes.

#### 2.1.3. Applying a Theoretical Model

The self-stigma theory [16,17,18,19], literature search, and qualitative interviews exploring sub-dimensions were used to assess the theoretical relevance of the sub-dimensions for the CSS. Some previous research has been conducted regarding self-stigma of families, but early research focused primarily on the discovery of self-stigma occurring in families [16,17]. Mak et al. [18] first clarified the dimensions of self-stigma of families, calling it “affiliate stigma;” they demonstrated its effective, cognitive, and behavioral dimensions [18]. After that, Mitterra et al. developed the Family Stigma Instrument with reference to Mak et al. [18]. The dimensions measured were “perceived family stigma”, “affective affiliate stigma”, “cognitive affiliate stigma”, “behavioral affiliate stigma”, and “positive aspects of caregiving”. Mak et al.’s scale had been developed for research in Hong Kong and China [18], and critical examinations of the scale’s items indicate that it is unsuitable for use in a Western context and it has limited applicability for people of different ethnicities [19]. However, as an overview, it is appropriate to refer to Asian research as closely as possible when conducting research in Japan. Hence, we decided that it was appropriate to examine our self-stigma construct in reference to the three dimensions used by Mak et al. 

The dimensions from the literature search and sub-dimensions from qualitative interviews were mapped to the self-stigma theory components (affective, cognitive, and behavioral) [18], and all were found to be theoretically relevant. Among the dimensions that were revealed by the literature search, negative prejudice and social rejection corresponded to the cognitive aspect of the self-stigma theory, emotional reactions corresponded to the affective aspect, and isolation and stigma management corresponded to the behavioral aspect. Among the sub-dimensions revealed in qualitative interviews, awareness of negative gazes corresponded to the cognitive aspect of the self-stigma theory, emotions arising from negative gazes corresponded to the affective aspect, and self-regulatory behavior to avoid negative gazes corresponded to the behavioral aspect. Social misunderstanding was not previously found in the self-stigma theory for patients with mental illness and their families, but it became apparent in our study—from the literature search and qualitative interviews—that these were sub-dimensions that were specific to families with ASD.

Based on the above, social misunderstanding, awareness of negative gazes, emotions arising from negative gazes, and self-regulatory behavior to avoid negative gazes were considered to be constructs of the CSS.

#### 2.1.4. Pilot Testing and Validating the Scale

The inductive item generation method was applied, as it is recommended when designing and developing new measures [38]. The items were created with reference to an interview with a spouse of a person with ASD, a review of previous studies on the self-stigma of families with ASD, and the self-stigma theory. The first author generated an extensive item pool, which was inspired by interview data and the self-stigma theory. According to Messick [39], expert judgments, such as the judgments of readability, ambiguity, and irrelevance of items, also play a critical role. The first author developed items iteratively in collaboration with the remaining authors and discussed them.

We pilot-tested the 30 items in collaboration with four of the partners of persons with ASD. Moreover, we obtained information regarding items that were difficult to answer as well as how long it took to answer them. Based on this information, to examine the content validity of the CSS, four people—including a scale-creation expert and experts on partners of persons with ASD—examined the items while using the Delphi method. This method seeks to reach the correct response through consensus. The scale-creation expert is a professor of social science with extensive experience in scale development. Moreover, the representatives of the self-help group have a wealth of experience with partners of persons with ASD. 

There were 30 items at the stage of trial production, but we deleted 16 items after having the parties confirm them because the target person was burdened or the content was duplicated. The process of correction and confirmation was repeated until we obtained consent from all members, resulting in 14 final items. Responses were given on a four-point Likert scale, ranging from “not applicable at all” to “very applicable”.

### 2.2. Participants

#### 2.2.1. Recruiting

We conducted our survey with the cooperation of two independent self-help groups for partners of persons with ASD. The main difference between the two groups was the representative and location. In each location, the group from which we recruited participants was the only group in the field of spouses/partners of persons with ASD. We explained the study’s purpose to the representatives of each self-help group, and recruitment was undertaken with those who met the eligibility criteria. We distributed the questionnaires with consent from the study participants at the self-help group. Anonymous self-administered questionnaires were distributed at the self-help group and returned by mail. The 400 questionnaires were distributed from August 2018 to December 2018 at a self-help group meeting. Responses were obtained from 259 people (recovery rate: 64.8%), of which 253 were included in the analysis. Self-help group 1 (SHG1) had 117 participants; self-help group 2 (SHG2) had 136 participants. The two groups were independent of each other.

#### 2.2.2. Inclusion and Exclusion Criteria

The sample inclusion criteria were women who (1) were partners/spouses of people with ASD or had been previously married to an individual with ASD and (2) had difficulties in their lives being married to/partners with individuals with ASD. In Japan, it was difficult to find a large sample of participants, as there are few organizations actively supporting adult partners of people with ASD. We were seeking spouses or partners of people with ASD, so it was not possible to recruit through a hospital or other public institution. Self-help groups for spouses/partners were the only available resource for finding participants who met our criteria, although, this source, inherently, greatly limited the number of potential participants for our research. Based on the limited opportunities available for recruiting participants, we limited our research to women, because the prevalence of ASD is higher in men, and most of the participants in the self-help groups being offered were women.

The sample exclusion criteria were as follows: the self-help groups were targeted for the partners and families of persons with ASD. In addition to the women partners/spouses who responded, two male spouses of persons with ASD, three children of persons with ASD, and one parent of a person with ASD answered. Based on the inclusion criteria, the study analysis excluded those six individuals who were not women spouses/partners of people with ASD.

### 2.3. Measures

#### 2.3.1. Self-Stigma of Families

We used the original version of CSS, which was a 14-item measure comprising four self-stigma sub-dimensions (social misunderstanding, awareness of negative gazes, emotions arising from negative gazes, and self-regulatory behavior to avoid negative gazes), in order to assess self-stigma. Respondents rated their level of agreement with each item on a four-point Likert-type scale (strongly disagree to strongly agree); higher scores indicated greater self-stigma.

#### 2.3.2. Quality of Life (QOL)

We used the World Health Organization Quality of Life-BREF (WHOQOLBREF) scale in Japanese [40]. The WHOQOLBREF is a 26-item scale that measures overall QOL and general health. It also measures four distinct QOL domains: physical health, psychological health, social relationships, and environmental aspects over the course of two weeks. All of the items were constructed on variations of a five-point Likert scale, with scores ranging from 1 to 5. We calculated an average score, as recommended by the scale creators.

#### 2.3.3. Participant Attributes

Each participant’s’ age, socio-economic status, marital status, and partner/spouse characteristics were included in analysis. Socio-economic status indicators used were highest level of education—junior high school or high school, junior college or technical school, or university degree or higher—and employment status—full time, part-time, or non-working. We obtained data on participants’ years of marriage, number of years since they noticed their spouses’ ASD, marital status, and reasons for marriage to determine marital status. Spouse status included the characteristics of spouses/partners with ASD using the brief Autism Spectrum Disorder in Adults Screening Questionnaire (ASDASQ) for adults with ASD [38]. The first nine items are scored as 0 (“no”) or 1 (“yes”) and collapsed to provide a summary symptom score (range 0–9) [41]. 

### 2.4. Ethical Considerations

First, we obtained the consent of two representatives of self-help groups. The two representatives checked the content of the questionnaire for ethical issues. After reviewing the questionnaire, the two representatives provided written consent indicating their approval of the content. Next, we distributed questionnaires and explained the content and purpose of the study in writing and verbally to obtain the consent of participants. The participants were informed that their responses were voluntary, and the questionnaires would be returned by mail. If the participants wished to withdraw from the study, they were free to do so. The confidentiality and security of the returned questionnaires were strictly controlled, and they were only used for research presentations. The data were statistically processed to guarantee all participants remained anonymous. Completion and return of the questionnaires constituted participants’ consent. This research study was approved by the ethics committee of all participating institutes. This study was approved by the Ethics Committee of the Faculty of Medicine of the University of Tsukuba (Approval number: 1304) and by the Ethics Committee of Tokyo Gakugei University (Approval number: 230).

### 2.5. Statistical Analyses

#### 2.5.1. Descriptive Statistics

Descriptive statistics were calculated for the CSS, WHOQOL, participants’ attributes, marital status, and spouse status for each group. T-test, Mann–Whitney U-test, and Pearson’s chi-square test were conducted to confirm whether there were any differences in the socio-demographic characteristics of participants in SHG1 and SHG2.

#### 2.5.2. Validity

According to Messick [39], validity was divided into three or four types (i.e., content validity, criterion-related validity, predictive validity, and coexistence validity) in the early 1950s. However, all of these traditional validity types can be captured by applying the integrated concept, called construct validity. As a study on construct validity, we examined substantive validity, structural validity, and external validity [35]. 

#### 2.5.3. Substantive Validity

For substantive validity, the study conducted qualitative interviews to explore sub-dimensions and a literature review as well as applied a theoretical model. 

#### 2.5.4. Structural Validity

As a study of structural validity, adjusted item-total correlation, and exploratory factor analysis (EFA) were performed. Because we investigated two independent groups, we analyzed them in two groups to confirm the factor structure. Item distributions were assessed to consider the estimation method for the EFA. EFA was conducted to determine the clearest meaningful factor structure with the sample. We examined the 14 items as ordinal variables while using the robust maximum likelihood. For the factor analysis, we examined multiple methods to retain factors, such as eigenvalues, clinical significance, examination of scree plot, and parallel analysis. After specifying the number of factors, this number was fixed, and an EFA model was assumed. The following fit indices were considered: comparative fit index (CFI), root mean square error of approximation (RMSEA), and standardized root mean square residual (SRMR) [42]. Next, McDonald’s ω was calculated to examine internal consistency [43]. The ω is better than the Cronbach α coefficient as a practical solution to the pervasive problem of internal consistency estimation [44]. 

#### 2.5.5. External Validity

After confirming that the factor structures between the two independent groups were the same, the subsequent analysis was performed by combining the two groups because we aimed to measure spouse stigma ultimately across groups. For external validity, criterion validity was analyzed. Additionally, as in previous studies, we used the CSS total score because we aimed at using family self-stigma in the total score. We used the correlation coefficient with WHOQOL to examine the external aspect of our scale. Next, we conducted the Shapiro–Wilk statistic normality test for the CSS (14 items), QOL (26 items), and QOL domains. In the CSS and QOL subdomains, Pearson’s product-moment (Pearson’s r) correlation coefficient was used when normality was confirmed, and Spearman’s rank correlation coefficient was used when normality was not confirmed. Missing values were excluded from the analyses, and we set the statistical significance probability to 5%. Statistical analyses were performed with SPSS version 25.0 (IBM Japan Inc., Tokyo, Japan) and Mplus version 8 (Muthén & Muthén Los Angeles, CA, USA).

## 3. Results

### 3.1. Participants

Table 1 presents participant characteristics. Participants’ average score on the WHOQOL 26 was 2.8.

### 3.2. Substantive Validity

As a result of the pilot-test, it was suggested that we reduce the burden on respondents given that the number of items was large; thus, the duplicate items were extracted, and items were deleted with the help of the scale-creation expert. Additionally, for items that were found to be difficult to answer, the language was corrected to make the items easier to answer. The process of correction and confirmation was repeated until all four members’ agreement was obtained regarding the final 14 items.

### 3.3. Structural Validity

An EFA was performed on all 14 items for two groups. Changes in the eigenvalues were examined using the scree plot to determine the number of factors to be retained. The slope of the plotline showed a sharp drop after four factors. A better method for evaluating the scree plot is the use of parallel analysis, as it is one of the most accurate methods for determining the number of factors to retain. We drew the random eigenvalues and simulated the data line. This analysis shows that four factors in the “factor analysis” parallel analysis lie above the corresponding simulated data line, and four components in the “principal components” parallel analysis lie above the corresponding simulated data line. In our case, the last factor lies very distant from the line—for both the principal components’ extraction and principal axis extraction. These results suggest a four sub-dimension structure. We also considered the clinical significance and, as these items were all considered to be important for the construct, we deemed it inappropriate to exclude them. Next, we selected four factors and the EFA was repeated. Table 2 shows the results. As expected, all 14 items were divided into four factors. Factor loadings were over 0.39 for all 14 items in the two groups. For SHG1, the goodness-of-fit indexes were CFI = 0.982, RMSEA = 0.051, and SRMR = 0.024. For SHG2, the goodness-of-fit indexes were CFI = 0.963, RMSEA = 0.072, and SRMR = 0.030. Further, adjusted item-total correlations of the 14 items ranged from 0.257 to 0.676.

We considered using CSS for total scores, as in a previous study [18]. For the internal consistency of total scores, the McDonald’s ω for 14 items was 0.823 for SHG1 and 0.793 for SHG2. The ω for each factor was determined to examine the possibility of a sub-dimension similar to that stated in a previous theory [18], as presented in Table 2.

Since the factor structure between the two independent groups was the same, and the factor loading and ω had similar values, the subsequent analysis was performed by integrating the two groups.

### 3.4. External Validity

Regarding external validity, we examined our criterion validity. The correlation coefficient between the CSS’s 14-item total score and the WHOQOL’s 26-item average score was −0.341 (*p* < 0.001). There were significant correlations between the CSS-14 and all the domains of the WHOQOL (see Table 3). The higher the CSS-14 score, the lower the WHOQOL.

## 4. Discussion

This study aimed to verify the construct validity of the newly created CSS scale. To an extent, our results confirmed the construct validity of the CSS. Our scale included social misunderstanding, which had not previously been used in the affiliate stigma scale [18]. The results of our study suggest a new construct exists that is unique to the stigma the spouses of individuals with ASD. This construct is different from the self-stigma theory of families with people with mental illness, intellectual disability, and physical disabilities, thus adding new knowledge to stigma theory [16,17,18,19].

### 4.1. Participants

The characteristics of the subjects of this study are described. Generally, couples cannot participate in self-help groups when they are depressed or ill. The 400 people identified as potential participants in this study were spouses of individuals with ASD who have had trouble in the community and were in a state where they needed to go to self-help groups. Of the 400 potential participants, 259 responded. The respondents are distinguished by those who have room to breathe to answer the questionnaire. In other words, the characteristics of those who returned the questionnaires were limited to those who had recovered enough to answer questions about their spouses. However, it should be noted that the average WHOQOL 26 for general Japanese women in their 40s was 3.28 points [23], whereas the participants’ average score on the WHOQOL 26 was 2.8 points in this study. The subjects of this study had lower QOL when compared to the national average, despite their participation in the recovery group for spouses of individuals with ASD who had difficult experiences. The groups that were not targeted this time may present more severe conditions than the results that were obtained in this study indicate.

### 4.2. Substantive Validity

For substantive validity, it is said that the importance of conceptualization and development of an Initial Item pool conceptualization [35]. After extracting scale items, two representatives of the self-help group and the researchers examined the appropriateness of the scale items. We considered this evidence that the appropriateness of the 14 items was confirmed, given that we obtained the agreement of all involved.

### 4.3. Structural Validity

In the present study, we found a good fit for the four-factor structure of the self-stigma scale. Factor loadings were over 0.39 in all 14 items. In a factor analysis, items with a factor loading of less than 0.30 are considered to be irrelevant because the latent structural changes measured are less than 10%. Therefore, it is recommended to keep items with a coefficient load of 0.40 and above [45]. In our study, only item nine of SHG 2 was 0.39, but this value was infinitely close to 0.4. In addition, there are only three items for this factor, and excluding this item in theory was inappropriate, so we left it on our scale. For future research, we think that wording needs to be modified. For SHG1, the goodness-of-fit indexes were CFI = 0.982, RMSEA = 0.051, and SRMR = 0.024. For SHG2, the goodness-of-fit indexes were CFI = 0.963, RMSEA = 0.072, and SRMR = 0.030. These values indicate that the model fits well [42]. Evidence from the structural aspects supporting the validity of the scale’s construct [39] was confirmed for all factors. When compared with the self-stigma theory [18,19], the social misunderstanding construct has not been previously seen in the self-stigma theory, although, previous studies on self-stigma of families of individuals with ASD show that parents experience various social misunderstandings, including difficulties that are related to self-stigma [29,30]. Therefore, it was considered clinically important to include these sub-factors. In previous studies on self-stigma, awareness of negative gazes is considered a cognitive factor, emotions arising from negative gazes are considered as an emotional factor, and self-regulatory behavior to avoid negative gazes is a behavioral factor.

The overall value of McDonald’s ω for the 14 items was 0.823 for SHG1 and 0.793 for SHG2, which is generally considered to be acceptable [35,45]. It is said that ω were over 0.82 or higher is desirable in some studies with strict criteria [46]. SHG1 cleared this strict standard, but SHG2 was slightly below the standard, although it was generally acceptable. Future studies need to be conducted. In SHG1 and SHG2, there were no items in which ω significantly increased when any of the 14 items were removed, and these 14 items were considered to be appropriate. We believe that these results support the construct validity of the scale [35].

### 4.4. External Validity

We also examined criterion validity. The verification of the criterion aspects confirmed their relationship with QOL [20,21,47], and they are considered as theoretically related constructs [20,21,22]. As a result, the CSS was negatively correlated with QOL, as seen in previous studies [20,21]. In the self-stigma theory, as a result of internalized stigma, persons experience reduced self-esteem and self-efficacy, which might lead them to avoid pursuing life goals [24], which leads to lower QOL. For each domain, the correlation coefficient was significant. The correlation between CSS and “IV environmental” in the QOL was -0.350. If the self-stigma score was high, self-regulation of behavior was observed, resulting in it becoming impossible to enjoy leisure activities, connection with people, or opportunities for social participation. Similarly, “How satisfied are you with the conditions of your living place?” included spouses in a broad sense, and there was a tendency for the correlation to increase. Among the QOL areas, the correlation coefficient with “physical health” was -0.191. This correlation might be lower, because those individuals may have recovered just enough to participate in the self-help group, but exhibit low physical health. These results are consistent with our predictions and provide evidence of the criterion validity of our CSS.

In summary, this is the first preliminary validation of the CSS to assess self-stigma among the partners of persons with ASD. We found the CSS to be reliable as a valid preliminary measure of internalized stigma in this sample. To date, no study has considered the partners of persons with ASD. Moreover, few papers have developed self-stigma measures specifically for spouses of persons with ASD. Thus, this study is significant, as it makes it possible to measure the self-stigma of partners of ASD patients. However, subsequent work is needed with additional independent data collected to gain a full psychometric validation of the CSS. The validation of the CSS should also be conducted in samples with varying symptom severity.

### 4.5. Limitations

Our study has some limitations. First, we did not examine the confirmatory factor analysis of the CSS. Therefore, it is necessary to confirm the factor structure in another large sample in the future. Second, the sample was relatively small, which is suboptimal for the conducted factor analyses and may have affected the precision of our results. Third, our study recruited participants from self-help groups for spouses of persons with ASD. We faced limitations in our population sampling and were not able to use random sampling, as there are no large institutions or organizations with large pools of potential participants. Therefore, we conducted an exhaustive survey of the pool of potential participants we could find—people who participated in the self-help group meetings. However, such meetings only include family members with depressive and anxious symptoms. Care must be taken when targeting men or targeting family members who are not depressed or overburdened, because the items and factor structure may change. 

Although there are various limitations, it was possible to measure self-stigma by developing a scale for the couples of persons with ASD and involving the largest number of people in a study conducted in Japan on this topic so far. Our scale could contribute to a further understanding of the situation of the spouses.

### 4.6. Future Directions

The first next step is to confirm the scale’s factor structure while using confirmatory factor analysis based on supplementary data. Second, future studies using a larger sample size are warranted to corroborate our psychometric findings. Third, future research is necessary to try to understand the phenomenon for not only women with depressive and anxious symptoms, but also men and other family members.

## 5. Conclusions

Our results suggest that the CSS scale is a valid instrument to assess self-stigma in spouses of individuals with ASD. Researchers and professionals that are interested in assessing the stigma of spouses of individuals with ASD can use our scale. Overall, our research serves to contribute to the self-stigma theory.

## Figures and Tables

**Table 1:** Socio-demographic characteristics of participating spouses of people diagnosed with autism spectrum disorder (ASD) (N = 253).

		Self-Help Group 1 (SHG1)		Self-Help Group 2 (SHG2)			*p*
		N = 117		N = 136			
		Mean ± SD	Median	Mean ± SD	Median		
**Age (Years)**	(25–76)	49.3 ± 9.9	50	50.2 ± 9.3	50	(a)	n.s.
**Socio-economic status**		**N (%)**		**N (%)**			
Educational level	Junior high school or high school	12 (10.5)		22 (16.2)		(c)	n.s.
	Junior college or technical school	47 (41.2)		55 (40.4)			
	University degree or higher	55 (48.2)		59 (43.4)			
Employment status	Full time	29 (25.0)		26 (19.1)		(c)	n.s.
	Part time	43 (37.1)		61 (44.9)			
	Non-working	44 (37.9)		49 (36.0)			
**Psychosocial status**	**Number of items (N)**	**Mean ± SD**	**Median**	**Mean ± SD**	**Median**		
WHO QOL-BREF ^1^	WHOQOL26 (26)	2.8 ± 0.7	2.8	2.8 ± 0.7	2.8	(a)	n.s.
(range 1–5)	Ⅰ Physical health (7)	2.9 ± 0.8	2.9	2.8 ± 0.8	2.7	(b)	n.s.
	Ⅱ Psychological health (6)	2.7 ± 0.8	2.7	2.7 ± 0.8	2.7	(b)	n.s.
	Ⅲ Social relationships (3)	2.6 ± 0.9	2.7	2.6 ± 0.8	2.7	(b)	n.s.
	Ⅳ Environmental (8)	3.1 ± 0.7	3.1	3.0 ± 0.6	2.9	(a)	n.s.
	Overall QOL and general health (2)	2.4 ± 0.9	2.0	2.4 ± 0.9	2.0	(b)	n.s.
**Marital status**		**N (%)**		**N (%)**			
Marital status	Married/living together	83 (70.9)		96 (70.6)		(c)	n.s.
	Married/separation	18 (15.4)		29 (21.3)			
	Divorced	6 (5.1)		3 (2.2)			
	Others	10 (8.5)		8 (5.9)			
Type of marriage	Love marriage	94 (81.7)		105 (77.8)		(c)	n.s.
	Arranged marriage	12 (10.4)		16 (11.9)			
	Others	9 (7.8)		14 (10.4)			
**Duration of marriage (years)**		**Mean ± SD**	**Median**	**Mean ± SD**	**Median**		
		19.9 ± 10.7	20	21.2 ± 10.6	21	(b)	n.s.
**Spouse with ASD**		**Mean ± SD**	**Median**	**Mean ± SD**	**Median**		
ASDASQ ^2^	(0–9)	6.3 ± 2.0	6	6.3 ± 2.1	7	(b)	n.s.

Missing data were excluded. SD = standard deviation. (a) T-test, (b) Mann-Whitney U-test, (c) Pearson’s chi-square test. n.s.: not significant. ^1^ The higher the score, the higher the quality of life (QOL). ^2^ The higher the score, the stronger the ASD tendency.

**Table 2:** Structural validity of the Couples Stigma Scale.

Items of the Couples Stigma Scale	Self-Help Group 1 (SHG1)				Self-Help Group 2 (SHG 2)			
			Factor Loading	Item-Total Correlation			Factor Loading	Item-Total Correlation
	Mean	SD			Mean	SD		
**Social misunderstanding**								
How well do people understand you as the wife of a person with autism spectrum disorder (ASD)?								
1 About wife’s loneliness	2.44	0.69	0.99	0.541 ***	2.49	0.71	0.94	0.552 ***
2 About wife’s difficulties	2.33	0.72	0.81	0.523 ***	2.21	0.77	0.83	0.517 ***
3 Wife’s mental and physical condition	2.36	0.73	0.88	0.515 ***	2.30	0.78	0.84	0.534 ***
**Awareness of negative gazes**								
How did people around you interact with you once you came to know about your husband’s ASD?								
4 Treated with an unfair attitude	1.43	0.85	0.90	0.617 ***	1.26	0.75	0.85	0.673 ***
5 Was seen as a different person	1.61	0.85	0.82	0.676 ***	1.41	0.86	0.89	0.676 ***
6 Avoided close relationships	1.35	0.89	0.79	0.634 ***	1.19	0.80	0.80	0.642 ***
7 Believed that the wife is also responsible	1.99	0.93	0.44	0.548 ***	1.86	0.94	0.57	0.573 ***
**Emotions arising from negative gazes**								
Have you experienced any of the following?								
8 I regret having married my husband	2.42	0.85	0.91	0.415 ***	2.46	0.76	0.94	0.322 ***
9 I wish I had immediately noticed the characteristics of my husband’s ASD	2.47	0.77	0.60	0.477 ***	2.63	0.72	0.39	0.323 ***
10 I feel lonely, angry, and sad about not being able to communicate with my husband	2.85	0.42	0.49	0.257 ***	2.87	0.42	0.44	0.371 ***
**Self-regulatory behavior to avoid negative gazes**								
Do you have the following with people around you?								
11 Avoid family relationships	1.65	1.02	0.84	0.646 ***	1.64	1.12	0.72	0.669 ***
12 Do not talk about my husband	1.57	0.93	0.68	0.662 ***	1.48	1.04	0.90	0.603 ***
13 Not letting the husband meet others	1.37	1.03	0.62	0.662 ***	1.29	0.96	0.81	0.626 ***
14 Thinking about excuses for the husband’s ASD characteristics	1.28	1.02	0.66	0.574 ***	1.10	0.96	0.62	0.519 ***
**14 Total items of the Spouse Stigma Scale**	23.02	5.90			21.92	5.84		
ω of 14 Total items	0.823				0.793			
Fit Indices								
CFI	0.982				0.963			
RMSEA	0.051				0.072			
SRMR	0.024				0.030			

R: Invert scale; Factor loading: Exploratory Factor Analysis (EFA), Estimator is robust maximum likelihood; *** *p* < 0.001. ω: McDonald’s ω. 4-point scale: I, “strongly disagree” to “strongly agree”; II, “not understood at all” to “understood very well”; III/IV, “not applicable at all” to “very applicable”.

**Table 3:** External validity of the correlation between the Couples Stigma Scale and WHO QOL-BREF (N = 253).

		Mean ± SD	Couples Stigma Scale
**WHO QOL-BREF**	WHOQOL26 ^1^	2.8 ± 0.7	−0.341 ***
	Ⅰ. Physical health ^2^	2.8 ± 0.8	−0.191 **
	Ⅱ. Psychological health ^2^	2.7 ± 0.8	−0.250 ***
	Ⅲ. Social relationships ^2^	2.6 ± 0.8	−0.293 ***
	Ⅳ. Environmental ^1^	3.0 ± 0.7	−0.350 ***
	Overall QOL and general health ^2^	2.4 ± 0.9	−0.206 ***
14 Total items of the Couples Stigma Scale		31.5 ± 7.4	

** *p* < 0.01, *** *p* < 0.001; QOL = quality of life; SD = standard deviation. ^1^ Pearson’s correlation coefficient. ^2^ Spearman rank correlation coefficient.
